# Supplementary material for: Effects of manipulating slowpoke calcium-dependent potassium channel expression on rhythmic locomotor activity in Drosophila larvae
Source: PeerJ. 2013 Mar 26;1:e57. doi: 10.7717/peerj.57 (PMC3628981; doi:10.7717/peerj.57)
Supplement: Supplemental Information 1 — Comparing rhythmic locomotor activity recorded from larvae dissected using the dorsal-midline or off-midline dissection. [file peerj-01-57-s001.pdf]

Supplemental Text S1

Erin C. McKiernan[1*]

[1]Instituto Tecnológico y de Estudios Superiores de Monterrey, Xochitepec, Morelos, México
[*]Correspondence: emck31@gmail.com

Dissection method alters timing of rhythmic motor activity in WT

Intracellular recordings were made from muscles 1 or 2 (M1, M2) in dorsal midline (DM) and off midline (OM) dissected larvae, as schematized in Fig. 1. Rhythmic activity was recorded in all 20 DM dissected larvae (100%) and in 25 of 27 OM dissected larvae (93%). Both A to P and P to A waves were observed in each group, with the latter the more prevalent type of activity. In the DM group, 4 of 20 larvae (20%) displayed A to P waves, while all 20 larvae (100%) displayed P to A waves. Similarly, A to P waves were recorded in 5 of 25 active OM larvae (20%), while P to A waves were recorded in all 25 (100%).

Of the 23 total bouts of activity recorded in DM larvae, 3 (13%) consisted of exclusively A to P waves, 18 (78%) of only P to A waves, and 2 (8%) were combination bouts comprised of both types of activity. In OM larvae, a total of 24 bouts were recorded, 20 (83%) of which were comprised of P to A waves, and 4 (17%) of which were combination bouts including both wave types. In the OM group no bouts of exclusively A to P waves were recorded. In both groups, individual P to A wave bouts ranged from 1 to 9 minutes. Combination bouts lasted 2 minutes in DM larvae, and 4-9 minutes in OM larvae. In sum, preparing larvae using our new OM dissection did not affect the incidence or duration of rhythmic activity, relative to larvae prepared using the DM dissection ($p > 0.05$).

The motor patterns recorded in DM and OM dissected larvae were quantified for comparison. Activity was quantified in 18 out of 20 active DM larvae, and in 21 of 25 active OM larvae. Fig. 1C shows representative recordings of P to A wave bouts in DM (top trace) and OM (bottom trace) dissected larvae, and reveals that the motor pattern was noticeably faster in OM larvae. Comparing histograms of burst duration, cycle duration, duty cycle, and quiescence interval showed shifts in the relative frequencies of these measures for the two groups (Fig. 2).

Minimum, maximum, and quartile values for each measure from DM and OM recordings are summarized for comparison in Table 1. Note that for groups in which animals displayed multiple bouts (i.e. bursting separated by 1 minute or more), the maximum cycle duration and quiescence interval was ≥ 60 seconds (s). Taking the differences between the quartiles for each group revealed that burst durations were 4.99-6.36 s shorter in OM versus DM larvae, representing a 43-44% decrease. Cycle durations were also shorter in OM larvae by 3.95-5.35s (27-29% decrease). Bursting in OM larvae comprised less of each cycle than in DM larvae, resulting in duty cycles that were smaller by 0.15-0.17 (18-23% decrease) and quiescence intervals that were larger by 1.02-1.23s (20-29% increase). In sum, the motor pattern recorded from OM larvae was quantifiably distinct from that of DM larvae on all measures ($p < 0.001$).

Figure captions

Figure 1: Intracellular recordings of rhythmic motor activity in dorsal and off midline dissected WT larvae. Schematics of dorsal (**A**) and off (**B**) midline preparations. Cuts (dashed lines) were made up the midline, or to right of the midline, as indicated. Larvae were pinned and cleaned so muscles (rectangles) and central nervous system (solid black) were exposed. Muscles 1,2,4,6,7 and abdominal segments A5-A7 are labeled. The hemisegmental nerves exit the ventral nerve cord and innervate muscles in each segment. For clarity, not all muscles, segments, or nerves are pictured. **C:** Recordings from M1 in A5 during P to A waves in dorsal (top trace) and off (bottom trace) midline dissected larvae. Scale bar: 20 seconds.

Figure 2: Quantification of rhythmic activity in dorsal and off midline dissected larvae. Distributions of the relative frequencies of burst durations (**A.**), cycle durations (**B.**), duty cycles (**C.**), and quiescence intervals (**D.**) for dorsal (black) and off (white) midline dissected larvae.

Tables

Table 1: Measures of motor pattern in dorsal and off midline dissected larvae

dissection	measure	n	min(s)	max(s)	Q1(s)	Q2(s)	Q3(s)	p-value[a]
**DM**								
	burst duration	18	2.90	34.10	11.31	12.94	14.80	-
	cycle duration	18	7.27	≥60	14.61	16.22	18.28	-
	duty cycle	18	0.10	0.99	0.74	0.80	0.84	-
	quiescence interval	18	0.12	≥60	2.57	3.12	4.16	-
**OM**								
	burst duration	21	1.61	17.21	6.32	7.19	8.44	<0.001
	cycle duration	21	5.19	≥60	10.66	11.81	12.93	<0.001
	duty cycle	21	0.05	0.93	0.57	0.63	0.69	<0.001
	quiescence interval	21	0.59	≥60	3.63	4.35	5.18	<0.001

[a]Mann-Whitney U Test/Wilcoxon rank-sum test, compared to DM
